# Endovascular repair with a physician-modified fenestrated endograft to treat abdominal aortic pseudoaneurysm with Behcet’s disease: a case report

**DOI:** 10.1186/s13019-024-02523-2

**Published:** 2024-02-04

**Authors:** Wenzhuo Lian, Xitao Song, Liqiang Cui, Yuehong Zheng, Changwei Liu, Leng Ni

**Affiliations:** Department of Vascular Surgery, Peking Union Medical College Hospital, Chinese Academy of Medical Sciences and Peking Union Medical College, Beijing, 100730 People’s Republic of China

**Keywords:** Abdominal aortic aneurysm, Behcet’s disease, Endovascular aneurysm repair, Physician-modified fenestrated endograft, 3D image fusion guidance

## Abstract

**Background:**

Aortic involvement in patients with Behcet’s disease (BD) is rare, but it is one of the most severe manifestations. Open surgical repair of aortic aneurysm is challenging considering the high risk of postoperative recurrent anastomotic pseudoaneurysms and is associated with a much higher mortality rate. Recently, endovascular treatment has proven to be a feasible, less invasive alternative to surgery for these patients.

**Case presentation:**

We report a total endovascular repair of a paravisceral abdominal aortic pseudoaneurysm in a 25-year-old male patient with BD. The pseudoaneurysm was successfully excluded, and the blood supply of visceral arteries was preserved with a physician-modified three-fenestration endograft under 3D image fusion guidance. Immunosuppressive therapy was continued for 1 year postoperatively. At 18 months, the patient was asymptomatic without abdominal pain. Computed tomography angiography demonstrated the absence of pseudoaneurysm recurrence, good patency of visceral vessels.

**Discussion and conclusions:**

Endovascular repair using physician-modified fenestrated endografts is a relatively safe and effective approach for treating paravisceral aortic pseudoaneurysm in BD patients. This technique enables the preservation of the visceral arteries and prevents aneurysm recurrence at the proximal and distal landing zones, which are common complications of open surgical repair in these patients. Furthermore, we emphasize the importance of adequate immunosuppressive therapy before and after surgical repair in BD patients, which is a major risk factor for recurrence and poor prognosis.

## Background

Behcet’s disease (BD) is a chronic multisystem disease that can involve the mucous membranes, skin, eyes, gastrointestinal tract, joints, vessels and neurologic system. BD with involvement of the vascular system is called vasculo-Behcet’s disease, which affects up to 50% of BD patients [1]. Arterial disease, especially aortic aneurysm or pseudoaneurysm, is a relatively rare complication that is highly lethal due to the risk of aneurysm rupture. Open surgical repair of an aortic aneurysm caused by BD is challenging since the vessel walls of BD patients are inflamed and fragile, and recurrence of aneurysms leads to failures at anastomosis sites [2–4]. Endovascular repair has become an alternative approach to the treatment of aneurysmal manifestations in patients with BD [5]. Here, we report a BD patient with a paravisceral abdominal aortic pseudoaneurysm treated with endovascular repair using a physician-modified fenestrated endograft under 3D image fusion guidance.

## Case presentation

**Fig. 1:**
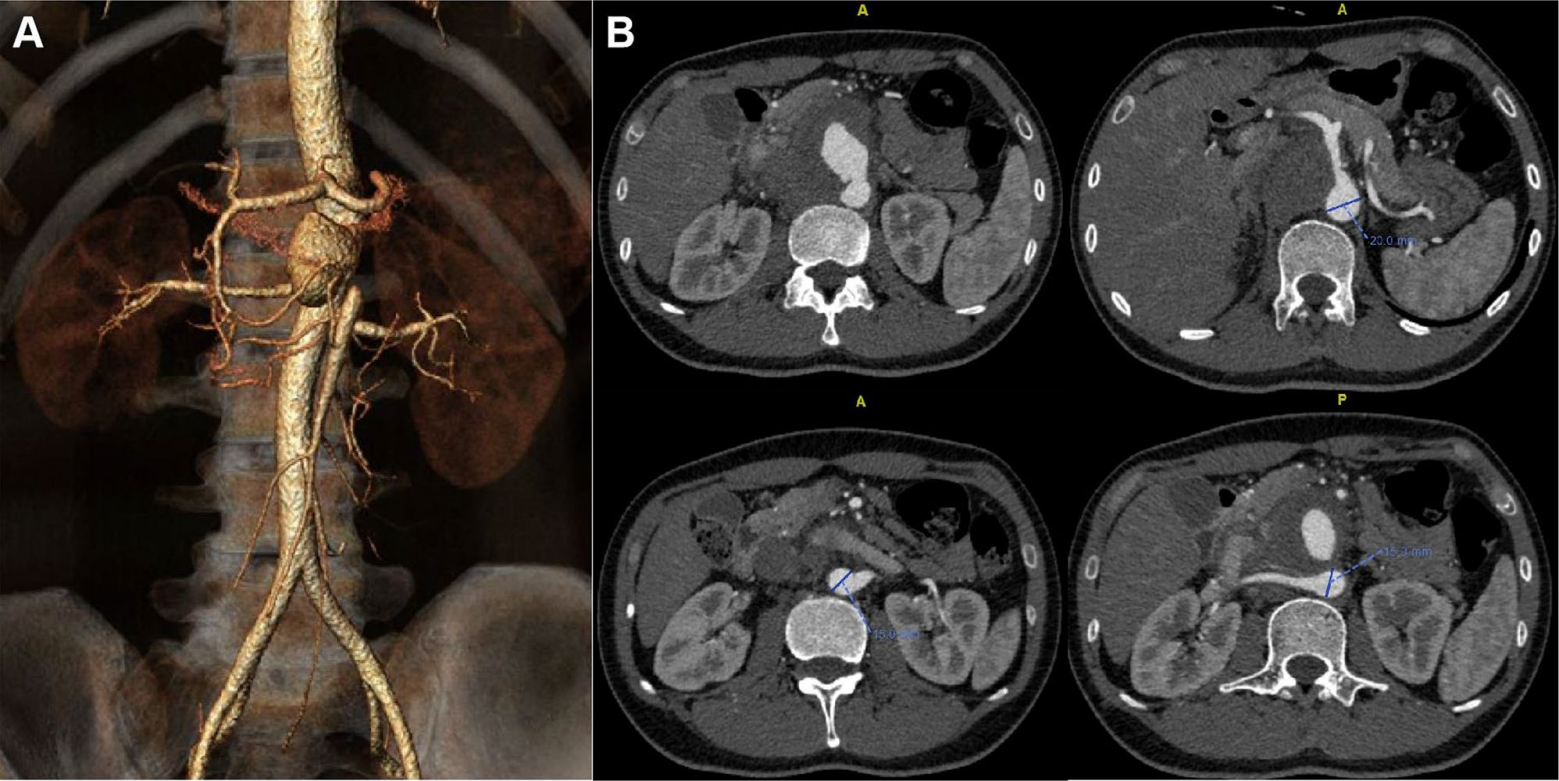
(**A**) Preoperative computed tomography angiography (CTA) with volume rendering reconstructions demonstrated paravisceral aortic pseudoaneurym. (**B**) Rupture location of pseudoaneurysm and aorta dimension at the level of visceral arteries

A 25-year-old man was transferred to our hospital owing to a clinical suspicion of abdominal aortic aneurysm. The patient reported persistent mild left upper abdominal pain for one month. He also had recurrent oral ulcers for 5 years 7–8 times each year. Four years earlier, he underwent carotid artery stenting due to rupture of a pseudoaneurysm of the right internal carotid artery, followed by pseudoaneurysm formation at the puncture site of the right femoral artery, and bypass surgery with a prosthetic graft was performed. He had genital ulcers along with nodular rash and folliculitis in both lower limbs for one year. A computed tomography angiography (CTA) performed prior to admission to our hospital demonstrated a 58-mm-diameter pseudoaneurysm located in his abdominal aorta at the level of the superior mesenteric artery (SMA). The proximal portion of the SMA was totally occluded due to compression of the pseudoaneurysm, while the distal runoff of the SMA was patent through collateral circulation from the CT and inferior mesenteric artery (IMA) (Fig. 1A). As measured by preoperative CTA, the average diameter of the aorta at 20 mm above the CT was 21 mm. At 20 mm below the right renal artery, the average diameter of the aorta was 17 mm (Fig. 1B).

The patient was diagnosed with vasculo-Behcet’s disease and received immunosuppressive medication for 3 weeks before surgical intervention. Methylprednisolone 40 mg combined with 560 mg of tocilizumab, later replaced by 30 mg of prednisone daily; 1 g of mycophenolate mofetil (MMF) twice daily; and 10 mg of methotrexate (MTX) weekly were administered. Laboratory findings showed a high sensitive C-reactive protein (hs-CRP) level of 10.87 mg/l (normal range: 0-8 mg/l) and an erythrocyte sedimentation rate (ESR) of 2 mm/h (normal range: 0-15 mm/h) before the operation.

**Fig. 2:**
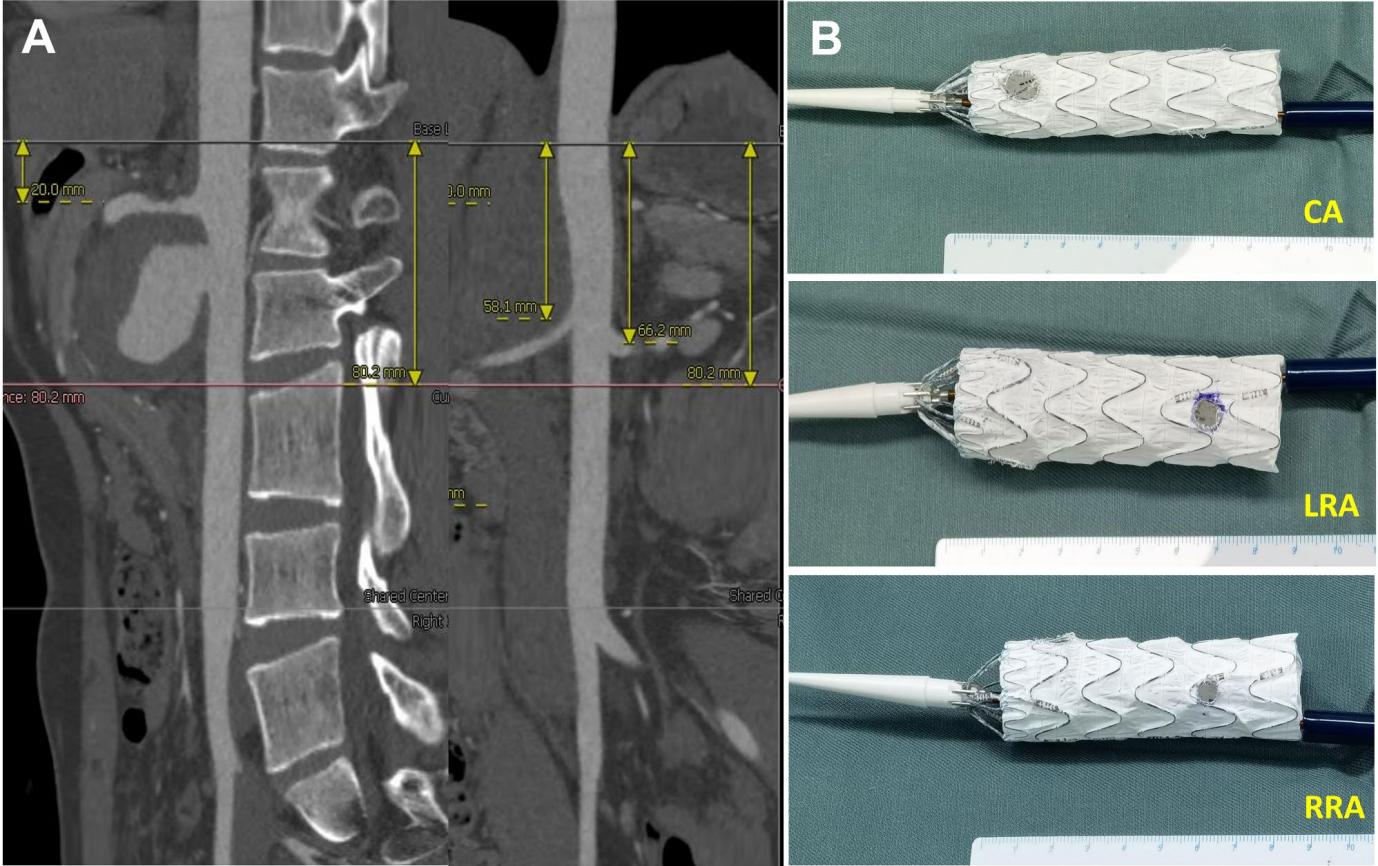
Physician-modified three-fenestration endograft was created based on centre lumen line reconstruction of the preoperative CTA. (**A**) centre lumen line reconstruction. (**B**) three fenestrations were created at planned positions for the celiac trunk (CT), left renal artery (LRA) and right renal artery (RRA).

Endovascular repair with physician-modified fenestrated endograft (PMEG) was planned for this patient. In order to prevent puncture site pseudoaneurysm through percutaneous approach in Behcet’s patient, we made surgical exposure of bilateral common femoral artery (CFA) and directly punctured the CFA and introduced the sheath. After a 22–80 mm Ankura cuff endograft (Ankura™, Lifetech, Shenzhen, China; 21Fr) was deployed on a sterile back-table in the operating room, three fenestrations were created according to perioperative planning: 8*8 mm at 15 mm from the proximal top of the endograft and in the 12:00 o’clock direction for the CT; 6*6 mm at 54 mm distal to the “o” mark of the stent graft and in the 9:00 o’clock direction for the right renal artery (RRA); and 6*6 mm at 60 mm distal to the “8” mark of the stent graft and in the 3:00 o’clock direction for the left renal artery (LRA) (Fig. 2). Fenestrations were reinforced by suturing metal coils (COOK Medical, Bloomington, IN, USA) with CV-6 Gore-Tex suture as radiopaque markers. Finally, the modified stent graft was re-sheathed into its original sheath by using 1 − 0 silk ties and silastic vessel loop to sequentially collapse the Z stents.

**Fig. 3:**
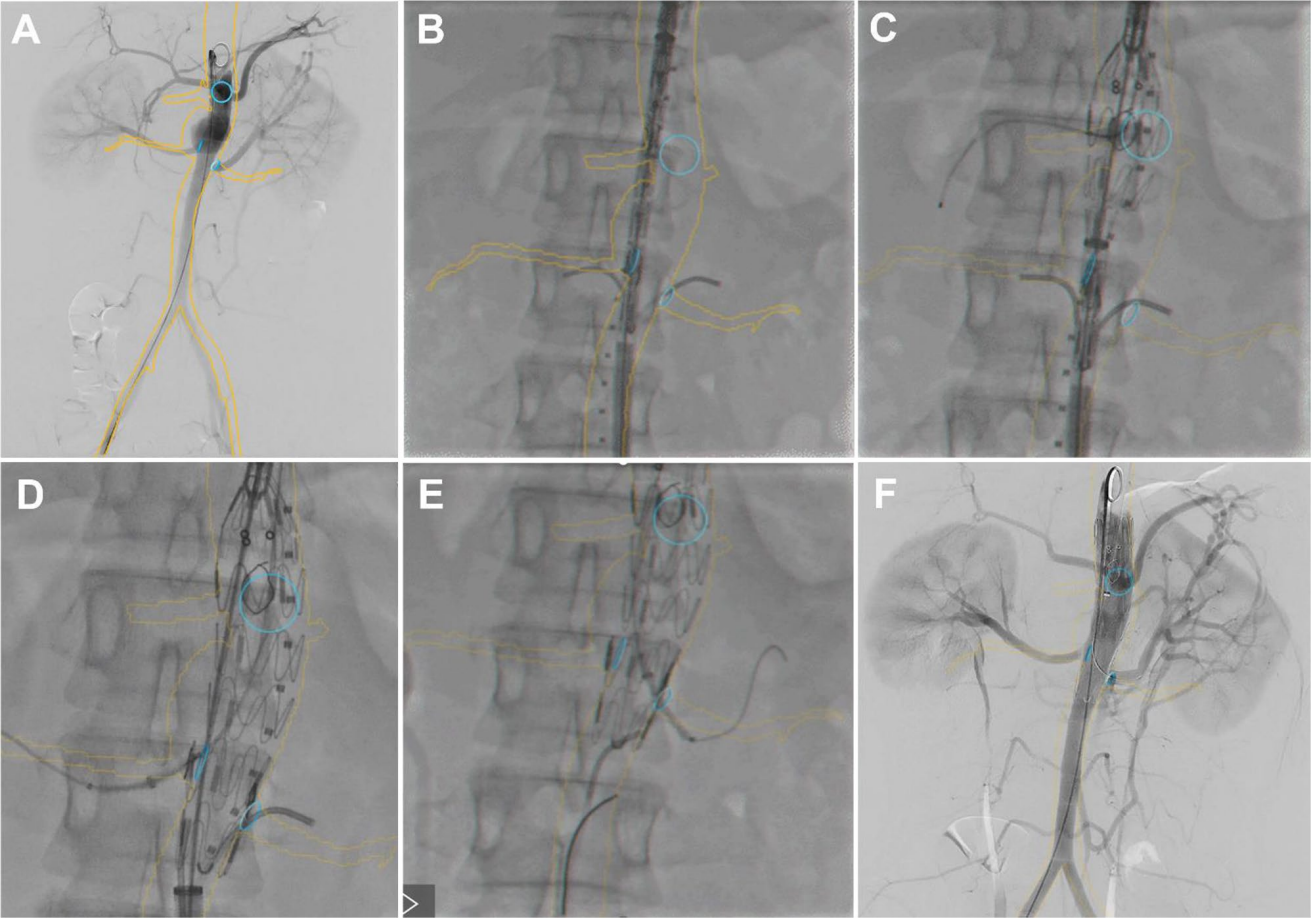
Intraoperative angiography based on image fusing views with landmarks at the ostia places of each visceral artery. (**A**) Preoperative angiography. (**B**) two Cobra catheters were placed in bilateral renal arteries for protection. (**C**) 5–40 mm balloon was delivered into the celiac trunk. (**D-E**) Viabahn covered stents in place in both renal arteries before deployment. (**F**) Final angiography showed the aneurysm was completed repaired with no endoleak and visceral arteries were patent

Before the procedure, bone and aortic 3D models were reconstructed from the preoperative CTA scan on a workstation (Advantage Workstation; GE Healthcare, USA). These models were then fused with live fluoroscopy (Fig. 3A). Two Cobra catheters were advanced through the left femoral artery into both renal arteries for protection (Fig. 3B). The PMEG was deployed from the right femoral artery. Accurate deployment of the PMEG and orientation of the hole towards the CT was guided by a 3D fused image. A 5–40 mm balloon (Powerflex, Cordis, USA) was delivered into the CT to facilitate adjustment of the PMEG for orientation of the holes towards the RAs (Fig. 3C). After successful cannulation of the two renal arteries from the left brachial artery, the two Cobra catheters were removed, and two 6–25 mm self-expanding covered stents (Viabahn, Gore, USA) were implanted in both renal arteries. The proximal ends of both stents were flared by a 10–40 mm balloon (Advance 35LP, COOK, USA) (Fig. 3D-E). Due to the big diameter of fenestration, bridging stent was not placed in CT. The PMEG was completely deployed, and the delivery system was retrieved. Completion angiography demonstrated that the pseudoaneurysm was completely excluded, and no endoleak was observed. The CT and RAs were patent, and perfusion of the distal segment of the SMA through collateral arteries was satisfactory (Fig. 3F). The total procedure time was 150 min, fluoroscopy time was 32 min, dose-area product (DAP) was 35.6 Gy.cm^2^, intraoperative bleeding was 100 mL, and the contrast volume used was 80 mL.

After endovascular treatment, the abdominal pain of this patient was obviously relieved. He received immunosuppressive therapy with 40 mg of intravenous methylprednisolone daily perioperatively and changed to 25 mg of oral prednisolone daily after discharge. MMF and MTX treatment was continued with supplementation of calcium and vitamin D. In addition, he was given 10 mg Xarelto for one month and 100 mg aspirin for long term after procedure.

**Fig. 4:**
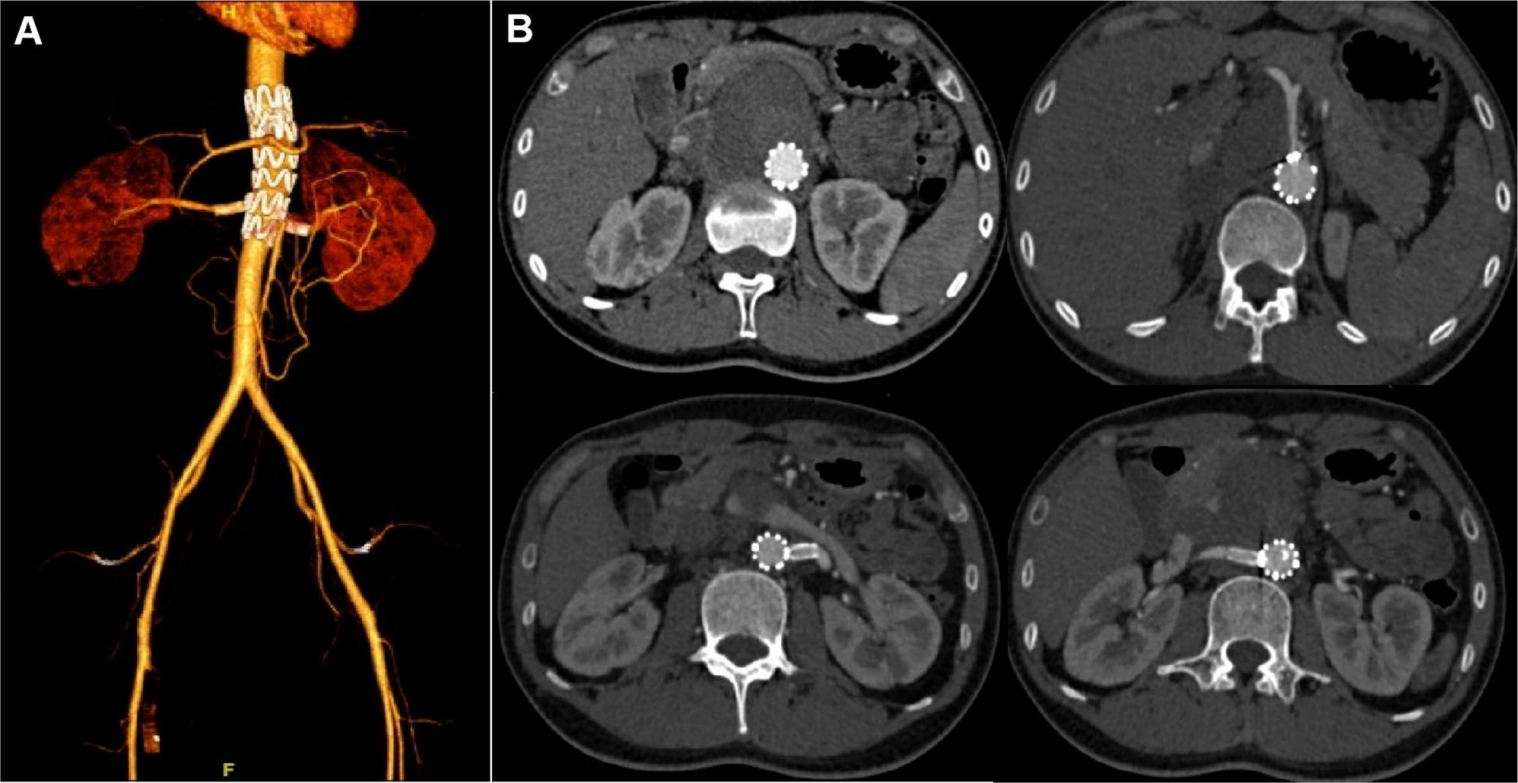
Follow-up CTA at 18 months with volume rendering reconstructions (**A**) and axial views (**B**) showed the aneurysm was completed excluded and the patent visceral arteries

At the 18-month follow-up, CTA demonstrated successful exclusion of the pseudoaneurysm and patent CT and RAs (Fig. 4). The distal portion of SMA is patent through collateral circulation as preoperative status. Oral prednisolone was continued and suggested to be reduced by 2.5 mg every 2 weeks. ESR and CRP levels were within the normal ranges.

## Discussion and conclusions

BD with aortic aneurysm is a relatively rare but severe complication with a high mortality rate. Immunosuppressive agents and surgery were recommended for the treatment of aortic aneurysms in BD by EULAR in 2008 and 2018 based on expert opinions [6, 7]. Some experts recommend that surgical intervention during the active stage of the disease should be avoided [8]. Furthermore, the updated 2018 recommendations suggest that medical treatment with cyclophosphamide and corticosteroids is necessary before surgical repair, but if the patient is symptomatic, emergent surgical treatment is advised without a delay [7]. Fortunately, severe spontaneous rupture of aortic pseudoaneurysms caused by BD is rare, and it is probably be due to inflammation of the peri-aneurysmal tissues and fibrotic reactions [9], which provides time for immunotherapy. Elevated ESR and hs-CRP of this patient at admission suggested the active stage of BD. Additionally, his abdominal pain was mild and stable in the past one month. Therefore, he was given immunosuppressive therapy first to stabilized the inflammation before surgical intervention.

Since some studies have reported that the recurrence rate following open surgical repair for BD aneurysms is approximately 30–50% [3], endovascular treatment with stent grafts has gradually become a reasonable alternative approach to avoid anastomotic pseudoaneurysms. Regarding the rupture site of pseudoaneurysm located adjacent to the offspring of visceral arteries, we planned physician modified three-fenestration stent graft to preserve visceral vessel patency. As previous report, we placed two Cobra catheters as landmarks for correct stent graft delivery and to preserve bailout parallel graft implantation in case of acute closure [10]. Compared with degenerative aneurysms, preoperative planning of endovascular repair for pseudoaneurysm caused by BD are somewhat different, including oversizing of stent graft, choice of proximal landing zone. Recurrent aneurysms occurring just proximal or distal to the stent graft were noted to have a recurrence rate as high as 40% [3]. Considering that the inflammatory process in BD not only affects the adjacent site of the aneurysm but also makes it difficult to locate a healthy vessel wall, the landing zone of the stent graft should be far away from the location of the aneurysm to prevent the recurrence of a new pseudoaneurysm. In this patient, although the rupture site of the pseudoaneurysm was 2 cm below the CT and 1.5 cm above the RRA, we chose 2 cm superior to the CT and 2 cm inferior to the RRA on the healthy aorta as the landing zone. In addition, the proportion of oversize stent grafts in treating BD aneurysms is commonly under 10% compared with 15–20% in treating degenerative aneurysms to reduce the irritation of stent grafts on the vessel wall, and stent grafts with lower radial force are also preferred [11]. In this patient, we chose a stent graft diameter of 22 cm because the aorta diameter at the proximal landing zone was approximately 20 cm. In this patient, the aorta is straight which facilitate catheterization of visceral arteries, preloaded guidewires and diameter-reducing wire are not necessary. Preoperative measurement and precise planning of fenestration is essential to ensure successful cannulation of the visceral arteries. In this patient, we used image fusion technology to mark the orifice of the visceral arteries preoperatively to facilitate visceral artery cannulation, avoid repeat angiography, reduce contrast agent usage and shorten the operation time.

For BD patients with vascular involvement, standard glucocorticoids (GCs) and immunosuppressive agents are important to limit pseudoaneurysm recurrence. The dosage of GCs should be sufficient at onset (e.g., 1 mg/kg prednisone daily) and gradually decrease to a stable level (e.g., 5–10 mg prednisone daily). Cyclophosphamide (CTX) is the first choice for immunosuppressive agents and is suggested for long-term intake. Monoclonal antibodies and TNF-α antagonists can also be used to alleviate inflammation [7]. Oral hormonotherapy can be replaced by intravenous methylprednisolone 3 days before the operation. Considering that BD is a chronic immune disease, GC and immunosuppressive agents should be continued for at least 2 years. Failure to maintain GC intake is a major risk factor for pseudoaneurysm recurrence in BD [12].

## Conclusions

Endovascular intervention with PMEG is feasible and effective for treating paravisceral aortic aneurysm in BD patients. In addition, this procedure could eliminate the potential risk for aneurysm formation at anastomotic sites in conditions of open surgical repair. However, experience with endovascular treatment in BD patients is limited, and long-term follow-up is required to further explore the safety and effectiveness of these interventions.

## Data Availability

All data and materials are available upon reasonable request from the corresponding author.

## Abbreviations

BD: Behcet’s disease
CTA: Computed tomography angiography
PMEG: Physician-modified fenestrated endograft
CT: Celiac trunk
SMA: Superior mesenteric artery
IMA: Inferior mesenteric artery
RRA: Right renal artery
LRA: Left renal artery
hs-CRP: High sensitive C-reactive protein
ESR: Erythrocyte sedimentation rate
MMF: Mycophenolate Mofetil
MTX: Methotrexate
GC: Glucocorticoids
CTX: Cyclophosphamide
DAP: Dose-area product
